# Extracellular Vesicle Profiling and Their Use as Potential Disease Specific Biomarker

**DOI:** 10.3389/fimmu.2014.00413

**Published:** 2014-09-01

**Authors:** Henrike Julich, Arnulf Willms, Veronika Lukacs-Kornek, Miroslaw Kornek

**Affiliations:** ^1^Department of Medicine II, Saarland University Medical Center, Homburg, Germany; ^2^Department of General, Visceral and Thoracic Surgery, German Armed Forces Central Hospital, Koblenz, Germany

**Keywords:** extracellular vesicles, microparticles, microvesicles, profiling, miRNA

## Abstract

Cell-derived vesicles in particular extracellular vesicles (EVs) such as microparticles (MPs) and microvesicles besides exosomes are raising more and more attention as a novel and unique approach to detect diseases. It has recently become apparent that disease specific MP signatures or profiles might be beneficial to differentiate chronic liver diseases such as non-alcoholic fatty liver disease and chronic hepatitis C, to monitor their progression or possibly to assess treatment outcome. Therefore EVs might serve as a novel inexpensive and minimally invasive method to screen risk patients for the outbreak of a disease even before the initial symptoms, to follow up treatment complications and disease relapse. The purpose of the current review is to summarize already published EVs signatures for a limited number of exemplary diseases and to discuss their possible impact. Additionally, it will be discussed if the combination of EV profiling and miRNA profiling could be a future joint tool for the purpose of detecting cancer and from far larger interest to ultimately distinguish among various tumor entities. EVs might increase the chance of early detection of chronic diseases or cancers especially if applied as part of yearly health screenings in the future.

## Introduction

Extracellular vesicles (EVs) were first time described as an unwanted contamination of an experimental preparation of platelets and eventually called “platelets dust” by Wolf (1). Advanced methodologies such as fluorescence activated cell sorting (FACS) enabled their detailed characterization during the last decade. The relatively novel term EVs includes *exosomes* and activation- or apoptosis-induced microparticles/microvesicles (*MPs*/*MVs*). MPs/MVs are 100–1000 nm in diameter, sometimes referred to *ectosomes* and representing a novel route of horizontal communication between cells within the living organism through various body fluids. In contrast, exosomes are smaller in size, <100 nm, and opposite to MPs they are formed and stored within the cell before their release (2–4). MPs/MVs are generated through a process of cell membrane shedding called ectocytosis that is associated with the activation of the complement system C5b-9 complex or influx of Ca2^+^. Moreover, this process includes specific sorting of membrane proteins into the shedded membrane fraction of MPs/MVs and phosphatidylserine exposure during cellular activation or early apoptosis (4, 5). It seems that the activation of the parental cell might be the major release trigger of MPs *in vivo*, since 80% of the CD8^+^ T cell-derived MPs were positive for the T-cell activation marker CD25 (6). MPs/MVs seem to resemble their parental cells and share with them many characteristics, such as surface receptors, integral membrane proteins, as well as cytosolic proteins, some mRNAs, and even miRNAs (7–11). Above features make these vesicles very attractive to use as novel minimal invasive biomarkers (12, 13).

Many recent reviews summarized and discussed in depth their differences, how to use them as putative biomarkers, their underlying unique release mechanisms, and their impact on cell–cell communication (3, 6, 13–16). The purpose of this review is not trying to go one better, but rather to discuss useful MP/MV surface biomarkers highly specific for a selected panel of diseases. Surface markers of MPs are especially relevant, since flow cytometry has become the standard method of choice for characterizing and quantifying MPs/MVs and their specific subpopulations (12, 17–20).

Extracellular vesicles can interact with their microenvironment *in vitro* and *in vivo*, as shown for many examples of EV–cell interactions that affected transiently the recipient cells. These changes on the recipient cells manifested as gain of function, as rescue of function, or as enhancing of function (4, 7, 8). Despite of these above effects on the microenvironment, EVs were and are utilized to detect pathophysiological changes and abnormalities in diseases under experimental conditions in plasma-, serum-, or other body fluids (12, 17, 19, 21, 22). Some publications quantified plain numbers of MPs/MVs under disease conditions vs. healthy controls (23, 24), whereas others determined a limited number of distinct MP/MV populations (<3) (18, 20–22, 25, 26). Because of the limited numbers of investigated MP/MV populations, the studies likely missed some interesting MP/MV populations that could be key for the particular disease. A better approach could be a comprehensive MP/MV based profile that could give the chance to discover other disease key players as reflected in the types and numbers of their associated MP/MV populations.

Additionally, among these early studies with a limited number of MP/MV populations, few groups acquired plain numbers of individual MP populations, using flow cytometry surface staining with or without Annexin V as a general MP/MV marker (25, 27). More importantly, the isolation protocol of these vesicles shows great differences from study to study. Thus, it might be difficult to assess the impact of these older data sets and to compare it in a meaningful way.

However, a new trend emerged to look for a wider panel of investigated MP/MV populations, pinpointing disease characteristics and providing evidence which MP/MV parental cell populations might play a role in disease outbreak and progression. Recently, MP based disease profiles of shared MP populations were provided for several human disorders such as atherosclerosis, arthritis, hepatitis C infection, non-alcoholic fatty liver disease (NAFLD), and malaria. In these studies, the authors have quantified several disease related and specific MP populations such as CD3^+^, CD4^+^, CD8^+^, CD11b^+^, CD14^+^, CD15^+^, CD20^+^, CD41^+^, and CD105^+^ MPs out of the heterogenic MP pool present within the circulation (12, 17, 19, 28, 29). Associated but not yet standardized MP/MV disease profiles consisting of individual selections of MP/MV surface marker that form a unique panel of EV surface antigens for each indicated disease are summarized in Table 1. Based on the existing MP/MV profiling data it seems that specific MP/MV marker combinations and their percentages as measured by FACS could be unique for each disease despite that several single markers appear to be associated with multiple diseases (such as CD3, CD4, and CD14, Table 1). Thus, the emphasis lies on the combination of MP/MV markers and their disease specific changes in these MP/MV populations that will determine specificity. Such MP/MV analyses should be further optimized and pursued for covering more disease-marker combinations as seen exemplarily in case of two gastrointestinal diseases (12).

**Table 1:** **Summary of the MP/MV profiles of indicated diseases and used surface MP/MV markers**.

Disease	MP/MV parental cell	MP/MV surface markers	Sample kind	Reference
**Meningococcal sepsis**	CD4 T-Lymphocytes	Annexin V + CD4	Plasma	(28)
	CD8 T-Lymphocytes	Annexin V + CD8		
	Monocytes	Annexin V + CD14	
	B-Lymphocytes	Annexin V + CD20	
	Platelets	Annexin V + CD61	
	Endothelial cells	Annexin V + CD62e	
	Granulocytes	Annexin V + CD66b	
	Erythrocytes	Annexin V + Glycophorin A	
**Atherosclerosis**	CD4 T-Lymphocytes	Annexin V + CD4	Atherosclerotic plaques	(29)
	Monocytes	Annexin V + CD14	
	Granulocytes	Annexin V + CD66b	
	Endothelial cells	Annexin V + CD144	
	Red blood cells	Annexin V + CD235a	
**Dermatomyositis**	T-Lymphocytes	Annexin V + CD3	Plasma	(21)
	Monocytes/Macrophages	Annexin V + CD14	
	B-Lymphocytes	Annexin V + CD19	
**Arthritis**	T-Lymphocytes	Annexin V + CD3	Synovial fluid	(17)
	Monocytes/Macrophages	Annexin V + CD14	
	Neutrophils	Annexin V + CD15	
	Platelets	Annexin V + CD41	
**Malaria**	T-Lymphocytes	Annexin V + CD3	Plasma	(19)
	Monocytes	Annexin V + CD11b	
	Platelets	Annexin V + CD41	
	Endothelial cells	Annexin V + CD105 + CD51	
	Red blood cells	Annexin V + CD235a	
**Chronic hepatitis C**	CD4 T-Lymphocytes	Annexin V + CD3	Serum	(6)
	CD4 T-Lymphocytes	Annexin V + CD4	
	CD8 T-Lymphocytes	Annexin V + CD8	
	Monocytes	Annexin V + CD14	
	Neutrophils	Annexin V + CD15	
	Platelets	Annexin V + CD41	
**Chronic hepatitis C vs. non-alcoholic steatohepatitis**	CD4 T-Lymphocytes	Annexin V + CD4	Serum	(12)
	CD8 T-Lymphocytes	Annexin V + CD8	
	Monocytes	Annexin V + CD14	
	Neutrophils	Annexin V + CD15	
	Platelets	Annexin V + CD41	
	iNKT cells	Annexin V + Valpha24/Vbeta11		

Upcoming sophisticated MP/MV based disease profile registers, based on standardized isolation and quantifications protocols, might give the upcoming opportunity to detect in a minimal invasive manner a wide variety of diseases using a single blood donation. In fact, one MP/MV surface marker selection (CD4, CD8, CD14, CD15, CD41, and iNKT) was able to discriminate between two chronic liver diseases (hepatitis C infection and NAFLD) and the corresponding healthy cohort. Thus, some disease parameters as serum ALT and histology were well reflected and correlated with the selected MP populations. MPs could in these cases be especially practical since histology based on liver biopsy, a tarnished gold standard, have high sampling variability as discussed by the authors (12). These data support the assumption of the underlying potential of monitoring and profiling EVs in future clinical applications.

**Figure 1:**
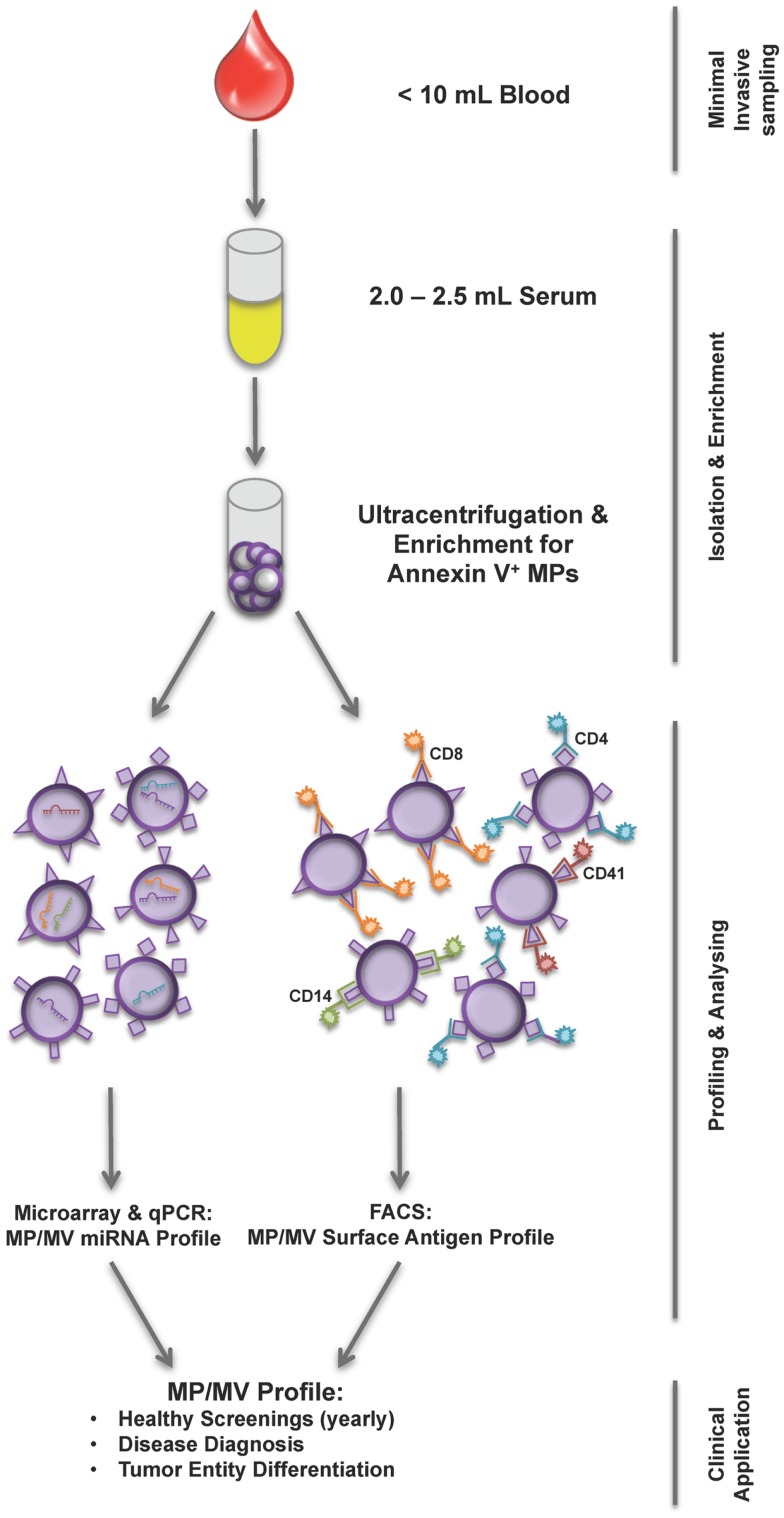
**Workflow of current MP/MV profiling strategies**. From less than 10 mL of blood sample first serum is prepared and subsequently MPs are isolated using ultracentrifugation and Annexin V enrichment. The isolation is followed by either a miRNA analysis of MP/MV content or FACS phenotyping using various surface marker combinations determining the underlying disease. The MP/MV profile generated this way could help health screening, diagnosis, and tumor differentiation.

Another and an even more doable application might be screening treatment response and outcome by EV profiling. Ideal would be a normalization of the EV disease profile toward an EV profile of healthy subjects during a positive treatment response separating responders from non-responders. Additionally, still speculative, a negative treatment response mirrored by an unchanged EV profile would give the chance to re-review treatment strategy. In accordance with these hypotheses, Shao and colleagues showed that MV protein typing, not EV profiling by surface markers, was successfully used as a predictive metric of treatment-induced changes (30). Nevertheless, how EVs will be characterized, if by their content or by their surface markers (Figure 1), EV isolation, their identification, and quantification must be simplified and standardized. Recently, a first step has been done toward simplifying MPs isolation by using Miltenyi Annexin V beads to capture MPs in body fluids recovering them in equal numbers as done previously by differential ultracentrifugation (31).

## EVs Diagnostic Potential and Limitation in Cancer

EV-based cancer screening provides a huge potential in various applications, especially differentiation among cancer entities, via using just a blood sample. Several experimental studies were carried out to detect cancer traces by tumor derived EVs; as exosomes or MPs/MVs. *In vitro* the release of tumor derived EVs showed impact on the tumor escape (32) angiogenesis (33) and tumor invasiveness (34) indicating that tumor EVs might help to prime the formation of the metastatic niche as reviewed by D’Souza-Schorey and Clancy (35, 36).

Up to date, only a very limited number of surface markers were explored *in vivo* on tumor derived EVs. Some publications outlined that in cancer patients the EV load as seen in plasma or serum samples was elevated (23, 24, 37–39). During the years, some EV surface tumor markers have been identified under defined experimental conditions (37, 40). Elevated levels of CD95L expressing MVs have been found to be associated with oral squamous cell carcinoma (OSCC). However, CD95L expressing MVs have also been associated with pregnancy, pinpointing that the use of single surface markers is not sufficient to associate MVs with specific diseases (41, 42). In 2008, a hepatocellular carcinoma (HCC) pilot-study was published, showing that the levels of endothelial (CD31^+^/CD42^-^) and hepatocyte (HepPar) derived MPs in HCC liver transplant patients were altered after surgery and correlated with the clinical outcome (37). *In vitro*, the approach to differentiate between prostate and breast cancer cell lines based on their MV signature consisting of more than 10 surface markers, unfortunately, did not result in a clear separation of the above tumor entities (43). As shown for these two examples, many markers might be shared between various cancer types as seen on cell lines and on tumorous and non-tumorous tissues making the differentiation extremely challenging. Even a highly sophisticated methodology as MV protein typing could confirm the presence of cancer but not its differentiation despite the fact that this profiling at least could identify therapy responder (30).

While these approaches were not as promising as hoped to pinpoint the cancer entities, the discovery of highly sensitive and specific “pan-cancer-MV/MP” marker could be usable to screen for cancer outbreak, especially, when the conventional differential diagnostic tools would not yet indicate the presence of the tumor or to overcome limitations of certain diagnostic methodologies such as biopsies or X-ray imaging if frequently applied.

Thus, a specific EV surface antigen combination unique for an individual cancer entity and differing from other cancer derived EVs is due to be explored but apparently not yet identified leading to the likely pre-matured conclusion that such EV surface combination might not exist. In the past decade, EpCAM (CD326) turned out to be a useful marker to detect circulating tumor cells (CTCs) or tumor stem cells (44–47). Although they are rare, the optical detection of such EpCAM^+^ tumor cells as done with the CellSearch™ system is in clinical use for a limited number of cancer entities (44, 48, 49). This method depends on metastatic spread of tumor cells from the primary tumor side and free float of tumor cells in the circulation (49). In contrast to these scarce CTCs, EpCAM^+^ EVs might be larger in numbers, a possible multiplier in fact of EpCAM^+^ tumor cells *in vivo*. More importantly, EV shedding is not depending on metastatic spread rather than activation and apoptosis. Thus, these tumor EVs might reach the circulation (50) as CTCs and being not restricted to metastatic tumor spread (51). Fairly, it must be taken into account that non-tumorous cells shed potentially EpCAM^+^ EVs making discrimination between tumorous and non-tumorous EVs difficult.

## miRNA Carrying Cancer Derived EVs

Another promising methodology of detecting and identifying tumor entities is currently the screening for extracellular miRNA. Some milestones were achieved as reviewed and summarized by others (52, 53). These studies gave rise to the hypothesis that miRNA packed in EVs as detectable in body fluids might serve as a promising biomarker for various pathological conditions (53) including prostate cancer (54) and glioblastoma multiform (29) or to monitor pathological changes of biological processes regulated by miRNA (55). Going in line with these observations and suggestions, it has recently been shown that these miRNA and mRNA detected within the EV lumen potentially participate in modulating the tumor niche by enhancing angiogenesis (56) and endothelial cell proliferation *in vitro* (57). Another work highlighted that muscle loss during cancer development might be attributed to miRNA packed in EVs (58).

Of note, combining miRNA arraying of MP content together with the surveillance of specific tumor derived EVs, might offer a novel opportunity to overcome the above discussed limitations due to overlapping surface markers as seen for tumor derived EVs forging a clear differentiation among tumor entities nearly impossible. So far, several interesting experimental attempts acknowledged the idea to isolate and screen tumor derived EV miRNA. Promisingly, Sun et al. demonstrated that the combination of EV isolation and EV miRNA arraying might be feasible as a new type of combined tumor biomarker (59). Noteworthy, they used crude MV preparation, without the enrichment for Annexin V positive MVs or for other known MV populations positive for any of the previously discussed cancer markers such as EpCAM. Nevertheless, their presented data convincingly demonstrated that miRNAs were differentially expressed in MVs originating from HCC patients compared with chronic hepatitis B and normal control cohorts (59). Another interesting publication that could distinguish between healthy and cancer patients used a similar methodology, isolation of crude EVs and EV miRNA array, for cholangiocarcinoma (CCA) diagnosis (60).

In both trials, miRNA differences were explored in samples of tumor free cohorts vs. tumor samples of known cancer entities raising the question if this approach can distinguish between unknown tumor entities as well. Therefore, the combination of EV profiling and FACS based sorting or magnetic beads based enrichment of these potentially tumor derived EVs with miRNA screening might enable scientist to address two main questions: is a tumor present and if so what kind/entity does the tumor has. For example, EpCAM^+^ EVs together with miRNA array could serve as such tool in the future.

## Conclusion

Nevertheless, the incline of tumor derived EVs or miRNA levels detectable in these circulating EVs in patients at risk with a high probability to develop cancer might provide a novel tool with a huge clinical potential and impact. If reliable and proven cut off values with useful positive predictive values for tumor derived or disease associated EV profiles can be achieved, the MP profile could pinpoint the underlying tumor or disease entity and even it could overcome sample variability as often seen in case of biopsies. This could be combined with screening for tumor EVs packed with a specific set of miRNA as discussed above. This way, not only the disorder entity could be revealed, but treatment outcome could be monitored separating responders from non-responders by one minimally invasive blood sampling. Disease registers, containing standardized sets of disease signatures of EV surface markers, and other associated and standardized disease parameters, might be very well the future. But until that several questions are remained and need to be addressed. Specifically, larger studies are necessary, providing evidence that the differentiation among disease entities is reliably and consistently feasible. Additionally, larger panel of surface markers and thereby MP/MV populations need to be measured in a comprehensive manner during the various diseases. Certainly, the ultimate goal should remain: the early detection of various chronic diseases especially cancer and the distinguishing of cancer entities.

## Conflict of Interest Statement

The authors declare that the research was conducted in the absence of any commercial or financial relationships that could be construed as a potential conflict of interest.
